# A non-threshold region-specific method for detecting rare variants in complex diseases

**DOI:** 10.1371/journal.pone.0188566

**Published:** 2017-11-30

**Authors:** Ai-Ru Hsieh, Dao-Peng Chen, Amrita Sengupta Chattopadhyay, Ying-Ju Li, Chien-Ching Chang, Cathy S. J. Fann

**Affiliations:** 1 Graduate Institute of Biostatistics, China Medical University, Taichung, Taiwan; 2 Institute of Biomedical Sciences, Academia Sinica, Nankang, Taipei, Taiwan; National Institute of Environmental Health Sciences, UNITED STATES

## Abstract

A region-specific method, NTR (non-threshold rare) variant detection method, was developed—it does not use the threshold for defining rare variants and accounts for directions of effects. NTR also considers linkage disequilibrium within the region and accommodates common and rare variants simultaneously. NTR weighs variants according to minor allele frequency and odds ratio to combine the effects of common and rare variants on disease occurrence into a single score and provides a test statistic to assess the significance of the score. In the simulations, under different effect sizes, the power of NTR increased as the effect size increased, and the type I error of our method was controlled well. Moreover, NTR was compared with several other existing methods, including the combined multivariate and collapsing method (CMC), weighted sum statistic method (WSS), sequence kernel association test (SKAT), and its modification, SKAT-O. NTR yields comparable or better power in simulations, especially when the effects of linkage disequilibrium between variants were at least moderate. In an analysis of diabetic nephropathy data, NTR detected more confirmed disease-related genes than the other aforementioned methods. NTR can thus be used as a complementary tool to help in dissecting the etiology of complex diseases.

## Introduction

Genome-wide association studies (GWAS) constitute a powerful means for analyzing common variations with minor allele frequency (MAF) greater than 1–5% [1]. GWAS have identified risk alleles for a wide range of complex human diseases, such as diabetes [2], heart disease [3], and Alzheimer’s disease [4], etc. Despite many successes in identifying risk alleles, most associated variants discovered through GWAS pertain to relatively small-to-moderate increases in risk and do not account for the majority of heritability estimated for complex human diseases and traits. An estimated 60–80% of human diseases can be attributed to heritability, however GWAS have identified only 5–10% of this heritability. This continues to lead researchers to contemplate which alleles underlie the missing heritability [5–7]. Many reasons have been posited for this shortfall in accounting for heritability [5,6,8]. A plausible explanation is that rare variants (with MAF < 5% or < 1%) are often not detected in most GWAS. Unlike common variants, which are usually found within intergenic or non-coding regions, most missense mutations are rare and expected to be harmful [9] and are thus expected to alter gene expression levels or change amino acid sequences, which could affect protein-protein interactions [10]. Furthermore, rare variants may have higher odds ratios (ORs); i.e., >2, compared with common variants (OR = 1.1–1.5) [11–13].

Although rare variants have been proven to contribute to certain complex diseases, they have not been discovered by genomic searches such as those for common single-nucleotide polymorphisms (SNPs) [14]. Many statistical methods that are currently used to detect disease-associated common variants have insufficient power to detect rare variants due to the relative large abundance albeit low frequency of rare variants [6,7,15–17].

Several methods can be used to detect rare variants within a gene, genomic region, or biochemical pathway, including the burden test and variance-component test, which assign weights to variants based on linear modeling of variant effects. Madsen and Browning [18] proposed a weighted sum statistic (WSS) method as one of the burden tests that assigns weights to variants according to their frequency in controls such that a variant with lower frequency would have greater weight. Li and Leal [19] proposed a combined multivariate and collapsing (CMC) method for case-control data as another burden test. One example of a variance-component method is the sequence kernel association test (SKAT) [20], which is advantageous when the effects of rare variants are in opposing directions or if they are comprised of a mixture of neutral and non-neutral effects. However, SKAT can be less powerful than burden tests if a large proportion of the rare variants in a region are truly causal and influence the phenotype in the same direction [20,21]. Hence, a variation of SKAT, termed SCAT-O, was proposed to maximize statistical power by using the data to optimally combine the burden test and the non-burden SKAT [22].

Some disadvantages exist in these burden tests for detecting rare variants. The performance of existing and well-established methods depends on the MAF threshold used to define rare variants, which can result in the inclusion of neutral variants or exclusion of causal variants in the analysis [23]. This problem can be exacerbated when both common and rare variants contribute to disease risk because common neutral variants are likely to be included when the MAF threshold is relatively high; hence, the power to detect an association could be diminished [18,19,23]. Furthermore, if the pooled common/rare variants are associated with the disease in different directions, i.e., some positively and others negatively, a few rare variant detection methods are very sensitive to the presence of protective and risk variants [24]. It’s likely the statistical significance could be diminished due to cancelation. Moreover, several studies have found that common variants may often have a key role as modifiers of the effects of rare variants in Mendelian diseases, thus it is reasonable to expect that this also holds true for common diseases [25]. Hence, combining information on common and rare variants is essential for identifying complex diseases.

Accordingly, we developed a method, the NTR (non-threshold rare) variant detection method, that does not require arbitrary frequency thresholds for collapsing alleles and accounts for the directions of effects to detect the combined signal from rare and common variants within a genomic region while properly accounting for linkage disequilibrium (LD) between variants. We were particularly interested in five factors that might influence power and type I error of our method: (i) different ORs; (ii) different MAFs; (iii) different LD between variants in each region; (iv) variations in noise within a region, i.e., the number of non-causal variants in each region; and (v) variations in direction within a region, i.e., the number of positive and negative variants in each region. To evaluate the validity of NTR, we compared the results to those obtained from CMC, WSS, SKAT and SKAT-O. By evaluating results from simulations, we addressed the advantages and disadvantages of applying these methods to detect associated rare variants. Finally, we performed a rare-variant analysis using those methods and a publicly available dataset of diabetic nephropathy (DN) which was downloaded from the Database of Genotypes and Phenotypes (phs000389). We compared the results with current knowledge of DN. Our results reveal discrepancies among methods for rare-variant detection. The information from our results will assist researchers in identifying biological links to the etiology of complex diseases.

## Methods

The common weaknesses and limitations of certain popular methods for detecting rare variants are their inability to account for LD within the region of interest, and the overestimation of the validity of any particular association for common variants. We therefore developed NTR, which integrates the effects of common and rare variants on disease occurrence into a single score and provides a test statistic for assessing its significance. NTR accounts for LD using Hedrick's multiallelic $D_{im}^{\prime }$ [26] (the range of $D_{im}^{\prime }$ is [0,1]) and gives more weight to genomic regions containing relatively more rare variants under the assumption that the effects of most rare variants are more deleterious than protective. Hedrick (1987) [26] proposed $D_{im}^{\prime }=\sum_{i}\sum_{m}p_{i}q_{m}|D_{im}/D_{max}|$, where *D*_*im*_ = *h*_*im*_ − *p*_*i*_*q*_*m*_, *h*_*im*_ denotes the population proportion of haplotype *A*_*i*_*B*_*m*_ for a two-locus haplotype consisting of alleles *A*_*i*_ and *B*_*m*_, while *p*_*i*_ = ∑_*m*_ *h*_*im*_ and *q*_*m*_ = ∑_*i*_ *h*_*im*_, the proportions of alleles *A*_*i*_ and *B*_*m*_, respectively. *D*_*max*_ is

$$min(p_{i}q_{m},(1-p_{i})(1-q_{m}))\;if\;D_{im}<0$$

$$min(p_{i}(1-q_{m}),(1-p_{i})q_{m})\;if\;D_{im}>0$$

In fact, a summary measure of gametic disequilibrium between two loci is often considered [27]. Therefore, in this study, we used $D_{im}^{\prime }$ which considers two alleles at each locus between the two loci, when there are only two alleles at each locus, there is a unique value of |*D*_*im*_/*D*_*max*_|. The range of $D_{im}^{\prime }$ is [0,1], independent of the *p*_*i*_ and *q*_*m*_, which makes cross-locus and cross-population comparisons uncomplicated [26,27].

For genome-wide data, such as whole-genome or whole-exon resequencing data, it is possible to categorize the data as “genes” or “genomic regions” and test the association of a specific variant with a disease/phenotype for each gene or region [28]. Let *Xij* be the number of minor alleles at the *i*th variant carried by the *j*th individual (both cases and controls) in a region (e.g., haplotype, gene, pathway), *i* = *1*, *2*, …, *L*, where *L* is the number of genotyped variants. We define the genetic score for the *j*th individual as follows:

$$S_{j}(k)=\left[\sum_{i=1}^{L}W_{i}X_{ij}+\sum_{i=1}^{L}\sum_{\begin{array}{c} m=2\\ m\neq i \end{array}}^{L}W_{im}(k)(X_{ij}+X_{mj})\right]\times PR$$

where *PR* is the proportion of rare variants (i.e., MAF < 0.01) among *L* variants and $W_{i}=|{log}_{2}{OR}_{i}|(\frac{1}{{MF}_{i}})$; *log*_2_*OR*_*i*_ is the logarithm of the corresponding OR (base 2) for the *i*th variant, and $\frac{1}{{MF}_{i}}$ is the reciprocal of MAF for the *i*th variant. The genetic effects of causal variants correlate inversely with their MAFs, and the ORs for causal variants have an exponential relationship with their MAFs [29]. Hence, we chose *log*_2_*OR*_*i*_ for use in this study. We calculated three different weight *W*_*im*_(*k*) values for interaction as follows.

$$W_{im}(1)=({log}_{2}{OR}_{i}+{log}_{2}{OR}_{m})\left(\frac{1}{{MF}_{i}}+\frac{1}{{MF}_{m}}\right)(1-D_{im}^{\prime }),$$

$$W_{im}(2)=|{log}_{2}{OR}_{i}+{log}_{2}{OR}_{m}|\left(\frac{1}{{MF}_{i}}+\frac{1}{{MF}_{m}}\right){(1-D}_{im}^{\prime }),$$

$$W_{im}(3)=(\left|{log}_{2}{OR}_{i}|+|{log}_{2}{OR}_{m}\right|)\left(\frac{1}{{MF}_{i}}+\frac{1}{{MF}_{m}}\right){(1-D}_{im}^{\prime })$$

These *W*_*im*_ values are the weights for the ith and mth variants, where $D_{im}^{\prime }$ is the Hedrick's multiallelic $D_{im}^{\prime }$ [26], which represents the degree of LD between the *i*th and *m*th variants. We give lower weights to SNPs in high LD since they carry redundant information.

The three different *W*_*im*_(*k*) values were designed to maximize the capacity to detect SNPs, and we selected the one weight that yielded the smallest p-value. The first term of *W*_*im*_(1) is the sum of two ORs, the first term of *W*_*im*_(2) is the absolute value of the sum of two ORs, and the first term of *W*_*im*_(3) is the sum of two absolute values of ORs. These three *W*_*im*_(*k*) values yield the same magnitude but can have different signs, thus avoiding cancelation of the results if two variants have different directions.

We use the sum of ranked scores from cases as the test statistic. In the formula for score *S*_*j*_(*k*), most of *Xs* (the number of minor alleles) equal zero, implying that most of *S*_*j*_(*k*) are also equal to zero. We then use the permutation strategy to assess the power and type I error rate since many tied ranks (i.e., when multiple scores = 0) exist, which could make the distribution of scores severely skewed. We adopted approach used by Sanat K. Sarkar et al., 1997 and Yoav Benjamini et al., 2001[30,31] that use false discovery rate (FDR) [32] for the multiple tests correction of the minimum P-value. The test is comprised of the following steps 1–3.

Step 1 All individuals (cases and controls together) are ranked according to their genetic scores, and the sum of the ranks for cases is calculated as
  $$R(k)=\sum_{j\in cases}rank\;(S_{j}(k)),k=1,2,3.$$
  *R*(*k*) is sum of independently and identically distributed random variables, and this is thus approximately normally distributed according to the central limit theorem.
Step 2 The affected/unaffected status is permuted among the individuals and repeated *n* times for samples $r_{1}^{\prime }(k),\ldots ,r_{n}^{\prime }(k)$ under the null hypothesis.
Step 3 The averages *m*(*k*) and sample standard deviations *s*(*k*) of $r_{1}^{\prime }(k),\ldots ,r_{n}^{\prime }(k)$ are calculated to yield the standardized score-sum *Z*(*k*) = (*R*(*k*) − *m*(*k*))/*s*(*k*). Under the null hypothesis, *Z*(*k*) follows an approximate standard normal distribution. *k* is chosen to produce the smallest p-value among three *Z*(*k*)′*s*. In other words,
  $$k^{*}=\underset{k}{\arg\;\min}\;P(Z>|Z(k)|)$$
  where *Z* is a standard normal random variable. Thus, the p-value is 2 × *P*(*Z* > |*Z*(k*)|).

### Simulation studies

We generated simulated data as in Basu and Pan (2011) [21,33]. A simulation study was undertaken using R software (http://www.r-project.org). Different scenarios were considered in order to explore the efficiency of NTR versus other methods in terms of LD, OR, MAF, noise (i.e., the number of non-causal variants in each region), and direction (i.e., the number of positive- and negative-acting variants) in each region. First, we generated a latent vector from a multivariate normal distribution with a first-order autoregressive covariance structure, e.g. there was a correlation between any two latent components. We used the correlation coefficient for LE (*ρ* = 0) and LD (*ρ* = 0.2, 0.4, and 0.6) within each region. In each region, we simulated eight causal variants and five different numbers of non-causal variants (0, 4, 8, 16, 32). We compared regions with only rare variants to regions with rare and common variants combined. Each rare variant had a MAF uniformly distributed between 0.001 and 0.01, and it was 0.01 to 0.5 for common variants. Second, the latent vector was dichotomized to yield a haplotype with MAFs selected randomly. Third, we combined two independent haplotypes and derived genotype data. Fourth, the disease status of the jth individual was generated from the logistic regression model. Fifth, as in any case-control design we sampled 3000 cases and 3001 controls in each dataset. Furthermore, we investigated additive genetic models and assumed two OR models: (1) only risk variants with OR in (1.2, 1.5); (2) risk and protective variants with OR in ((2.5, 0.4), (1.2, 0.8)).

## Results

### Type I error rate

**Fig 1:**
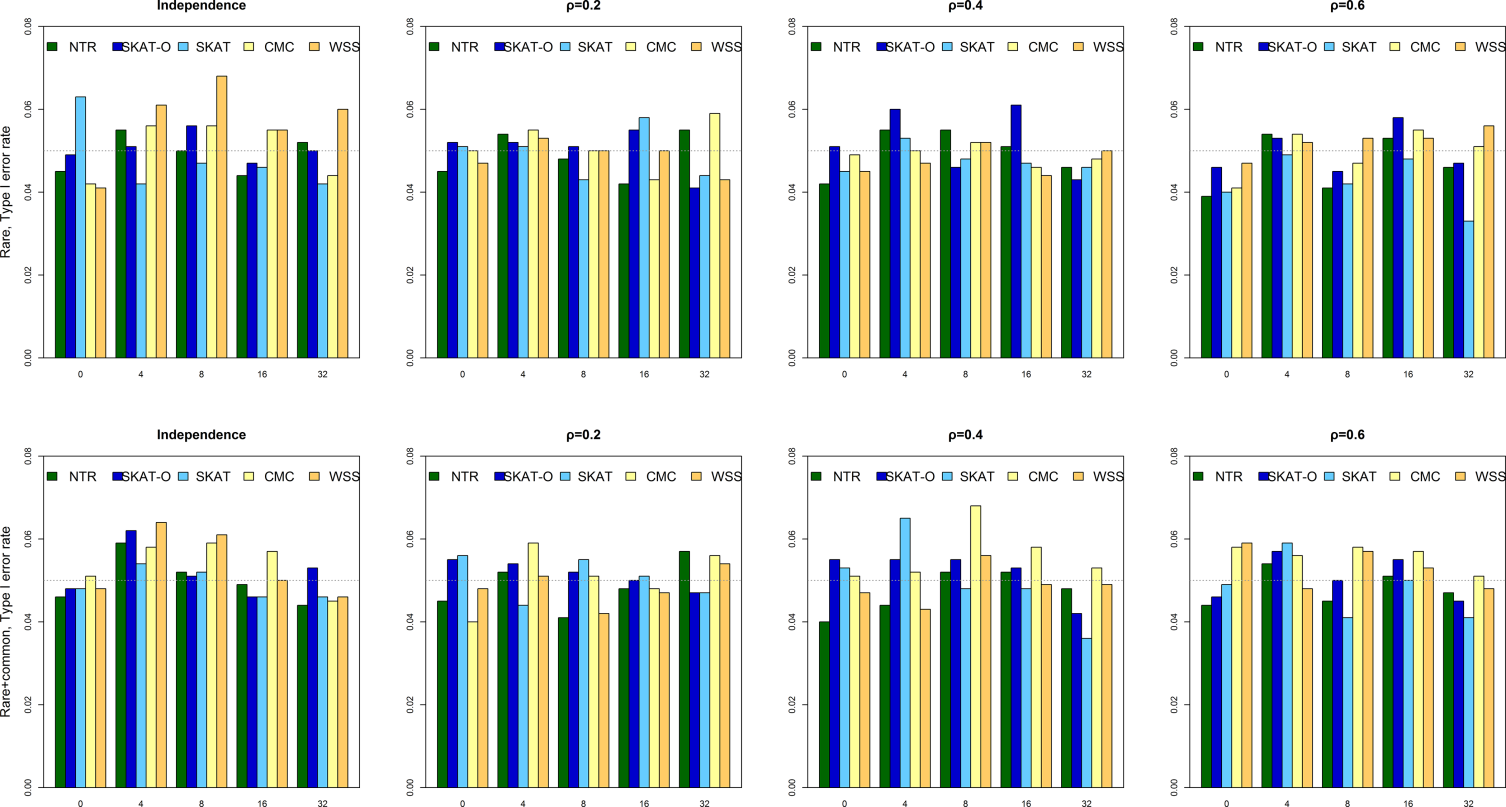
Type I error rates under different scenarios.

Fig 1 reports the type I error rate for each of the five methods in all scenarios, and it confirms that our simulation parameters were valid in the sense that each type I error rate matched the nominal significance level of 0.05. From the Fig 1 and S4 Table, we found that type I errors were controlled well.

### Power comparisons

To evaluate power, 1000 permutations were performed under each scenario. In total, there were 1000 simulations for power evaluation for each scenario. We considered five different amounts of non-causal variants (0, 4, 8, 16, 32) and four correlation coefficients (0, 0.2, 0.4, 0.6). As for OR, we considered two levels for a single direction (1.2, 1.5) and two levels for two opposing directions ((2.5, 0.4), (1.2, 0.8)). In our simulation, causal variants were not limited to being rare, as in reality, causal variants can be quite common. For these eight causal variants, in all scenarios and all simulated datasets, ~45% were rare (MAF < 1%), 62% were uncommon (MAF < 5%), and 38% were common (MAF > 5%).

**Fig 2:**
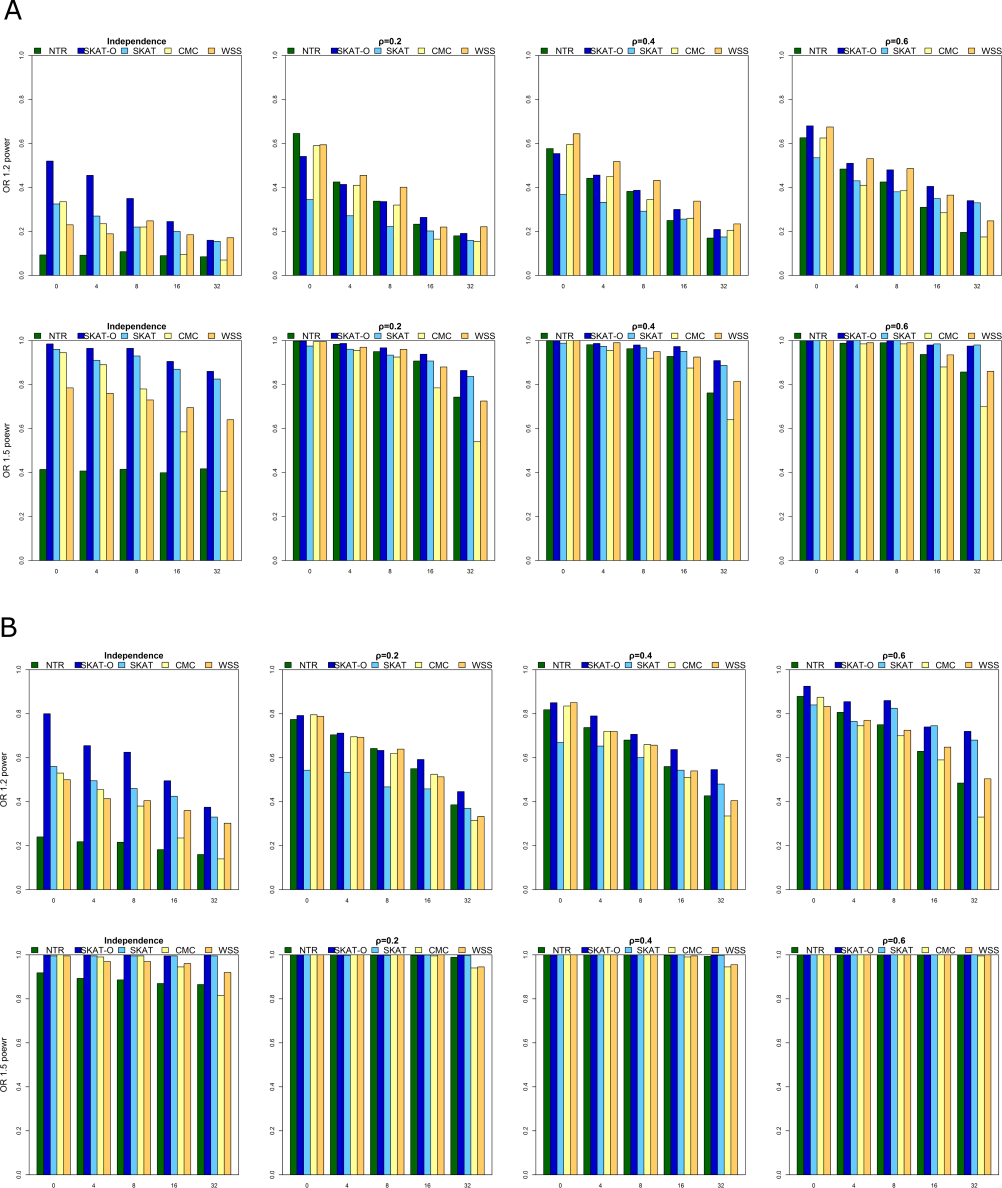
A: Power for rare causal SNPs when OR = 1.2 or 1.5. B: Power for rare and common causal SNPs when OR = 1.2 or 1.5.

**Fig 3:**
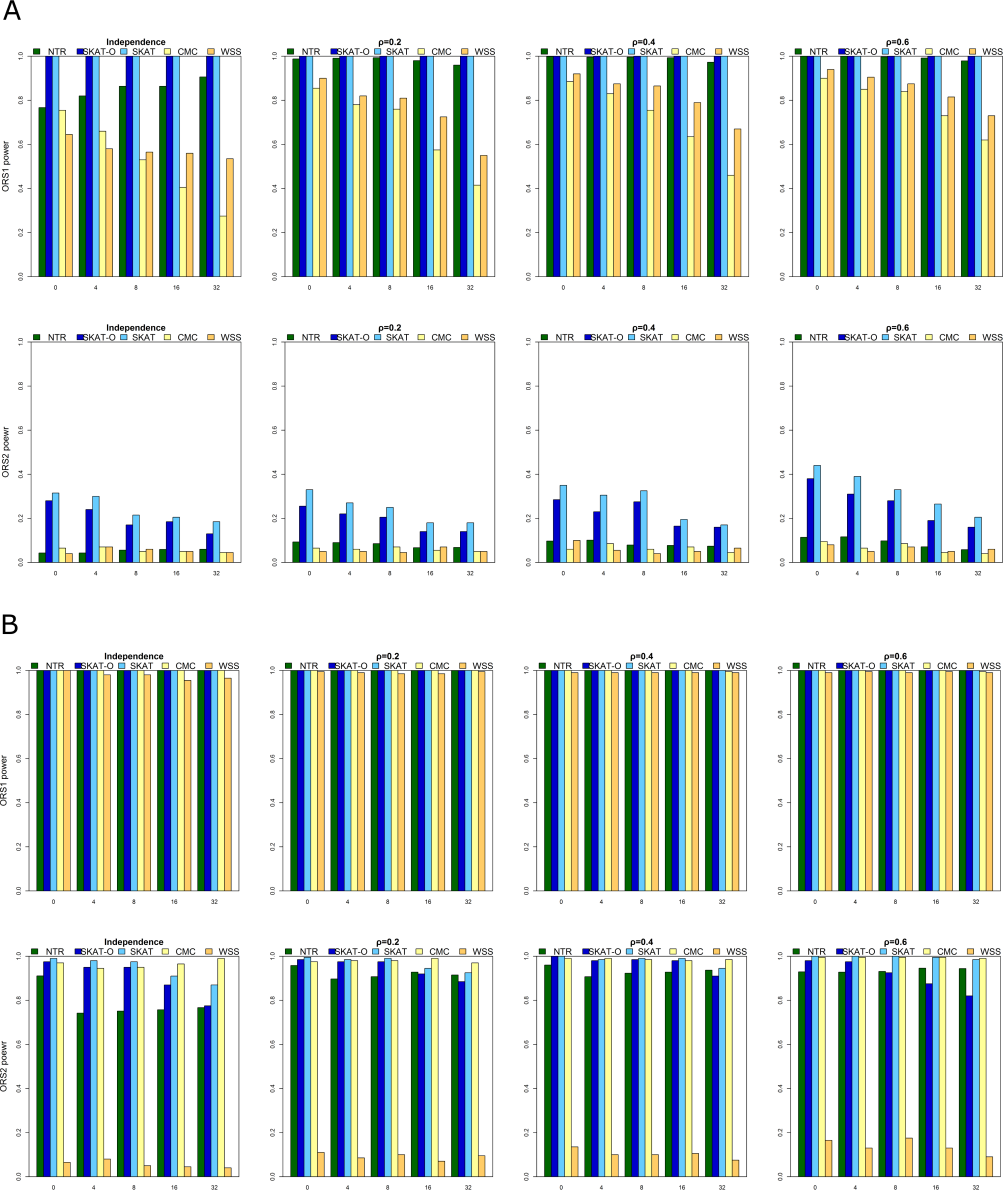
A: Power for rare causal SNPs under ORS1 and ORS2. B: Power for rare and common causal SNPs under ORS1 and ORS2.

Fig 2 presents power for rare causal SNPs (panel a) and for rare and common causal SNPs (panel b) in one direction (only risk variants). The two rows represent OR = 1.2 and 1.5, and the four columns represent the different correlation conditions. Fig 3 presents power for rare causal SNPs (panel a) and for rare and common causal SNPs (panel b) in two directions (risk and protective variants). The first row (ORS1) represents OR = (2.5, 0.4), and the second row (ORS2) represents OR = (1.2, 0.8). The impact of OR, LD, MAF (i.e., the proportion of rare and common variants in each region), noise (i.e., the number of non-causal variants in each region), and direction (i.e., the number of positive- and negative-acting variants) in each region on power was explored separately. All methods gave a higher power for larger OR, lower noise, larger LD, one direction, and regions that included both rare and common variants. In contrast, the scenario with OR = 1.2, non-causal variants = 32, and ρ = 0 was the worst-case scenario with respect to power rating, and all methods showed an ~80% decrease in power, i.e., to 20% (Fig 2A).

For one-direction scenarios (Fig 2A and 2B), NTR had higher power when ρ = 0.2 or ρ = 0.4 with OR = 1.2 regardless of the number of non-causal variants and causal variants with or without common variants than SKAT, CMC and WSS. In one of the scenario, e.g. OR = 1.2, ρ = 0.2, no noise and no common variants, NTR showed a 30% increase in power compared to that for SKAT (64.55% vs 34.5%) (Fig 2A). However, SKAT-O had the best performance when ρ = 0 with OR = 1.2 or 1.5. Under the remaining scenarios, NTR and SKAT-O had comparable performance.

For two-direction scenarios (Fig 3A and 3B), none of the methods could detect associations effectively for OR = 0.8 or 1.2 for causal variants without including common variants regardless of LD in a region. Under the remaining scenarios, NTR, SKAT-O, and SKAT had comparable performance. CMC and WSS were consistently the least powerful tests among the methods we compared, regardless of scenario. The power of CMC was greatly influenced by the number of non-causal variants in each region because of the collapsing-based method, which may be diluted by the number of non-causal variants increased in each region. Except for WSS, all methods showed an ~70% increase in power, to 98%, for two-direction scenarios (Fig 3A and 3B).

In summary, when the variants were independent, SKAT-O was more powerful; when the variants were correlated, NTR had the advantage of considering the effect of LD, thus making it more powerful. CMC and WSS were the least powerful methods regardless of scenario.

### Application of NTR to DN data

DNA from 1726 individuals (823 DN cases and 903 neurologically normal controls) in the UK-ROI collection (All Ireland and Warren 3 Genetics of Kidneys in Diabetes UK Collection) were genotyped using the Omni1-Quad array (Illumina, San Diego, CA, USA) downloaded from the Database of Genotypes and Phenotypes (phs000389.v1.p1). Chronic inflammation is a common contributor to progressive renal failure and leads to increased damage to mitochondrial DNA. Pathogenic mutations in mitochondrial DNA are an increasingly recognized cause of chronic morbidity, with mitochondrial mutations being implicated in a range of complex disorders, including kidney disease [34–36]. Hence, we focused on eight mitochondrial genes that E. J. Swan and coworkers reported as being associated with kidney disease [37]. Data for all SNPs in these eight mitochondrial genes were analyzed with the trend test. We then applied NTR, SKAT, SKAT-O, CMC, and WSS methods to detect rare variants. In addition, we collected 102 DN-related genes through the use of QIAGEN’s Ingenuity^®^ Pathway Analysis (IPA^®^, QIAGEN Redwood City, www.qiagen.com/ingenuity). We employed these 102 DN-related genes to confirm previous DN findings.

### Results for DN data

A total of 341 SNPs were located in the eight mitochondrial genes, and all SNPs satisfied the following quality-control criteria: genotype call rate < 0.95, departure from Hardy-Weinberg equilibrium (i.e., p-value < 10^−4^), and no cut-off allele frequency.

The results of the SNP analysis are shown in S1 Table. Of the 341 SNPs located in the eight mitochondrial genes, no significant SNPs were identified using a threshold of p < 1.4 × 10^−4^ (Bonferroni adjustments based on 341 SNPs). This was consistent with previous studies [37] that reported that no significant SNPs were detected in the DN data. Among them, rs1408705 (with borderline significance, p = 0.00024) is located in *PACRG* (PARK2 co-regulated), which was previously found to be deleted in clear-cell renal cell carcinomas [38].

By using CMC, WSS, SKAT, SKAT-O, and NTR, we assessed the association between rare variants for 7 mitochondrial genes (one of the 8 genes was excluded because it only contained one SNP). Among these 7 genes, CMC (3 genes) found more associated genes compared with NTR (1 genes), WSS (1 gene), SKAT (0 genes), and SKAT-O (0 genes) (Table 1). Among the 1 genes detected with NTR/CMC/WSS, *TOP1MT* (topoisomerase (DNA) I, mitochondrial) was found by NTR (p = 0.02557), CMC (p = 0.02054) and WSS (p = 0.01967).

**Table 1:** Summary of results for the detection of rare variants in the DN dataset.

Gene symbol	Chr*	Num**	NTR	SKAT	SKAT-O	CMC	WSS
*PACRG*	6	205	0.15337	0.226	0.36498	0.01396	0.8646
*TOP1MT*	8	9	0.02557	0.36791	0.38406	0.02054	0.01967
*COQ5*	12	6	0.58652	0.46948	0.4821	0.38591	0.73318
*GATC*	12	5	0.59337	0.71746	0.7179	0.16793	0.59031
*SPTLC2*	14	34	0.43911	0.07054	0.11769	0.0336	0.09501
*COX10*	17	41	0.98879	0.07526	0.12677	0.05307	0.88205
*TXNRD2*	22	40	0.15633	0.59836	0.764	0.37006	0.90084

*:chromosome.

**: the number of SNPs located within the gene.

Of the 102 DN-related genes that were collected by IPA, NTR found considerably more genes (9 genes) than SKAT (7 genes), SKAT-O (8 genes), CMC (6 genes), or WSS (8 genes) (see S2 Table). Among these 9 genes, the association with *GSS* (glutathione synthetase, p = 0.04), *CTSH* (cathepsin H, p = 0.0018), and *PPARG* (peroxisome proliferator activated receptor gamma, p = 0.0165) was found only by NTR (see S2 Table). Moreover, *NR1H3* (nuclear receptor subfamily 1 group H member 3) was found by all the methods except CMC. *NR1H3* belongs to the NR1 subfamily of the nuclear receptor superfamily and is highly expressed in visceral organs including liver, kidney, and intestine [39]. *NR1H3* also plays numerous roles in pathways involved in metabolic syndrome [40]. Finally, *PPARG* has been implicated in the pathology of numerous diseases including obesity and diabetes [41].

**Fig 4:**
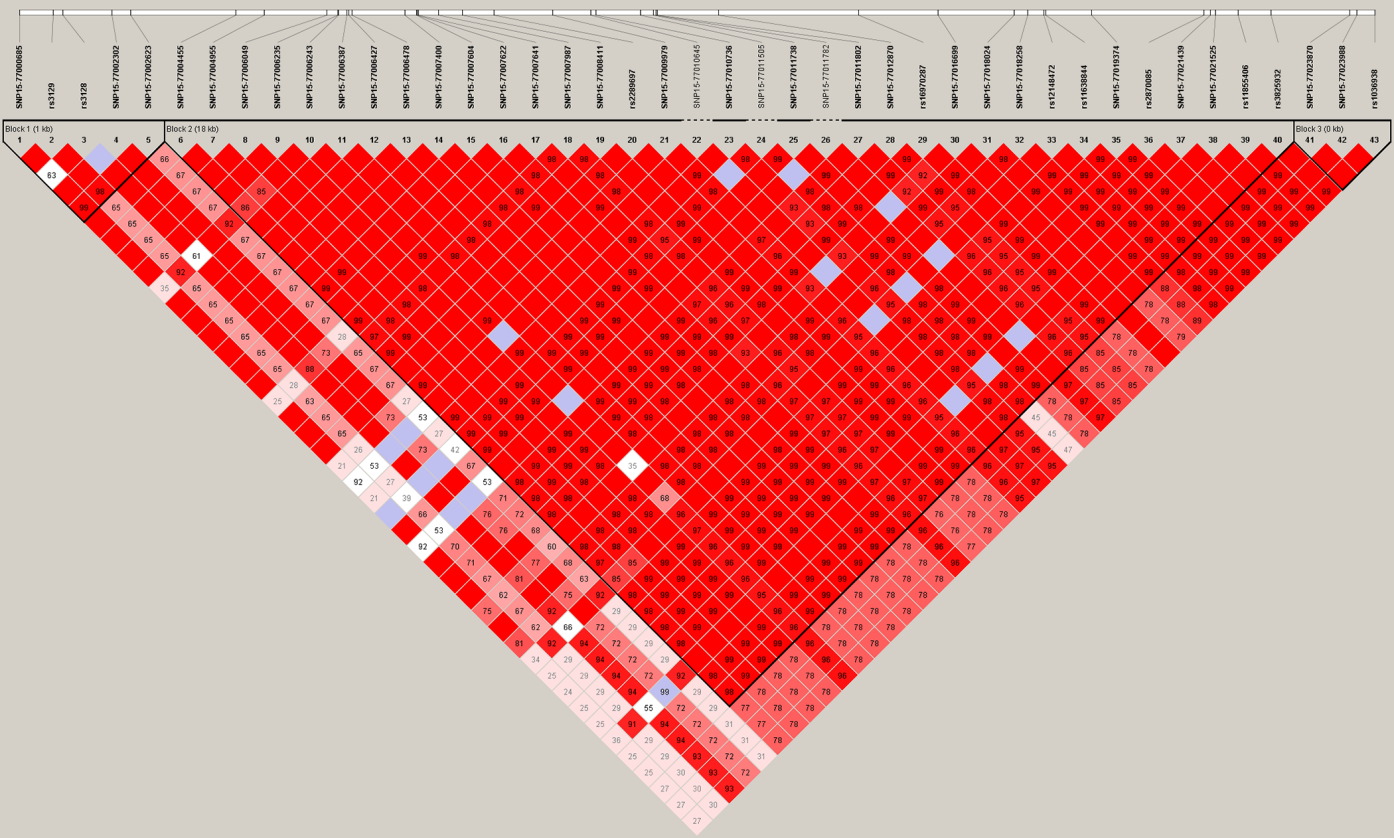
LD structure of *CTSH* in the DN dataset. The numbers in squares are D’. A standard color scheme in Haploview is used to display LD with bright red for very strong LD (LOD = 2, D' ≈ 1), white for no LD (LOD < 2, D' < 1), and pink (LOD = 2, D' < 1) and blue (LOD < 2, D' ≈ 1) for intermediate LD. [LOD, logarithm of odds].

**Fig 5:**
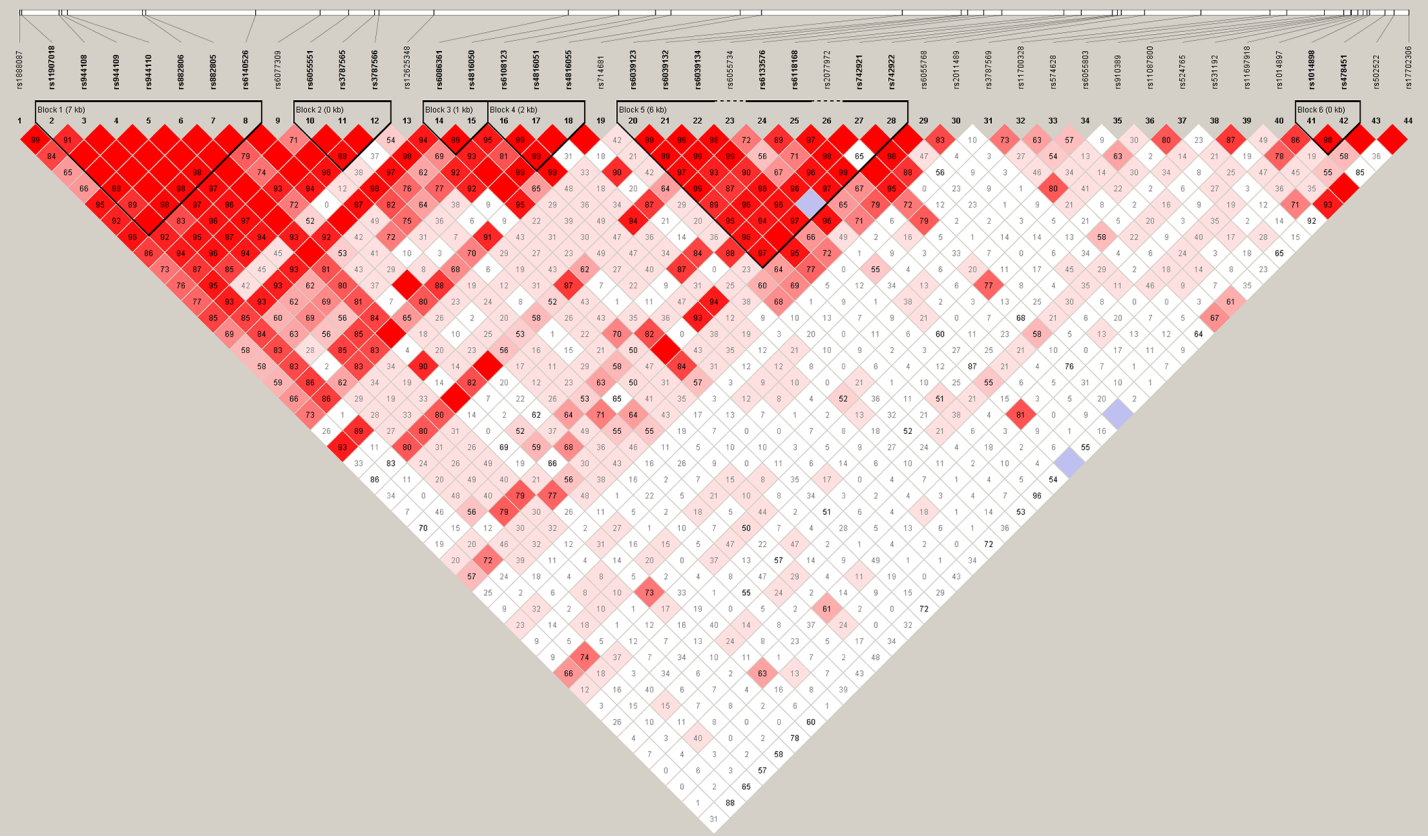
LD structure of *ANGPT4* in the DN dataset.

Our research illustrates the important role of rare variants in DN and shows that NTR is useful for analyzing real datasets. Therefore, we also examined the LD structure in the DN data. We found that most of the $D_{im}^{\prime }$ values of the significant genes for NTR are close to 1, whereas most of the non-significant genes for NTR are much lower than 1. For example, despite the similarly large number of SNPS for *CTSH* (43 SNPs, Fig 4) and *ANGPT4* (44 SNPs, Fig 5), LD for *CTSH* SNPs was greater and yielded a lower p value (p = 0.0018), and LD was smaller for *ANGPT4* and yielded a non-significant p-value (p = 0.4252). Strong LD and small NTR p-values were also found for *CR1*, *REN*, *NR1H3*, *GAS6*, and *GSS*, whereas low LD and large NTR p-values were found for *AKR1C3* and *CXADR*.

## Discussion

NTR was designed for detecting the combined signal from rare and common variants and does not require arbitrary frequency thresholds for collapsing alleles. Thus, the associations contributed by both common and rare variants are less likely to be overlooked. When using burden tests, it is usually necessary to remove all variants above a certain MAF threshold for the signal to not be overwhelmed by common variants. However, all MAF frequency thresholds are arbitrary. The signal from rare variants will be swamped by noise from common variants when the MAF threshold is too high. On the contrary, true causal variants may be neglected when the MAF threshold is too low. NTR utilizes information for both risk-associated and protective SNPs and considers LD among all variants within a genomic region. NTR efficiently weights all variants by combining the values of Hedrick's multiallelic $D_{im}^{\prime }$ and the reciprocal of MAF to improve the performance for detecting rare variants. NTR proved to be a flexible statistical method that can assess associations between phenotypes and rare and common genetic variants. High-density genetic maps built with SNP markers that are polymorphic in various genetic backgrounds are very useful for studying the genetics of traits as well as genome organization and evolution. High-throughput genotyping technologies, such as sequencing-based genotyping [42], have provided rapid, efficient, and cost-effective genotyping approaches that have proven their efficiency for the construction of saturated genetic maps and mapping of genes and quantitative trait loci in the human genome [43]. Therefore, NTR integrates the effects of LD into a single score to detect rare variants, thus making it indispensable for future genetic studies of complex diseases. In addition to using a stratification analysis before NTR, covariates could be cooperated into our proposed genetic score equation. The extensions will be carried out in the future.

In this study, we conducted extensive simulations to evaluate the performance of NTR for detecting rare variants. Simulation results demonstrated that the performance of NTR was superior to that of other methods over a wide range of scenarios, especially when the effects of LD between variants (Figs 2 and 3) were considered. In addition, the power of NTR was robust under all the five levels of the number of non-causal variants for both directions (Fig 3). In most scenario, SKAT-O performed best, however, the performance for NTR was better than that for SKAT-O while OR = 1.2 (Rare, Rare + Common, and number of causal variant were lower than 16). In addition, the performances of all methods were comparable when OR = 1.5 and ρ > 0.2 for causal variants while including common variants. Moreover, NTR had higher power when ρ = 0.2 and ρ = 0.4 with OR = 1.2 for causal variants including common variants than those obtained from SKAT, CMC and WSS (Figs 2 and 3). However, a mean value of *r*^2^ = 0.30 ± 0.32 has been observed for pairwise distances of <25 kb [44,45]. For common variants, very few had OR values >2, and most values fell between 1.1 and 1.4. For rare variants, many have OR values >2 [45,46]. As an example of a common disease, namely colorectal cancer, the highest overall OR ever reported was 1.22 [47]. In our analysis of DN data, NTR was more powerful than the other methods. Our one-direction simulation results (Fig 2) showed that SKAT-O performed better than SKAT. In our two-direction simulation results (Fig 3), however, SKAT-O was less powerful than SKAT. These findings are consistent with previous studies because SKAT was designed for detecting rare variants that have different directions [20,21]. Hence, SKAT was less powerful than SKAT-O when a large proportion of the rare variants in a region were truly causal and influenced the phenotype in the same direction. On the contrary, SKAT performed better than SKAT-O when the rare variants in a region influenced the phenotype in two directions. CMC and WSS were the least powerful among the methods we compared, regardless of scenario. The power of CMC and WSS was influenced by the number of non-causal variants in each region. Due to the nature of the collapsing-based method, power might be compromised when the number of non-causal variants increases in a particular region. As for the type I error rate shown in Fig 1 and S4 Table, the range of type I error for NTR was 3.9–5.9%, we found that type I errors were controlled well for the method.

The statistic of NTR is *R*(*k*), i.e., the sum of score ranks from cases, and we used a permutation strategy to calculate the corresponding p-value. In this regard, we used the Mann–Whitney U test, but the performance was insufficient in that an excess number of OR. Too many tied ranks could affect the accuracy of the results. In addition, we found that the power of Mann–Whitney U test in our simulation was proportional to the number of non-causal SNPs; hence, a greater number of non-causal SNPs would increase the power, but this is counterintuitive. The reason could be that a greater number of SNPs diversifies the rank scores, so the Mann–Whitney U test tends to yield smaller p-values. However, in simulations we found that the permuted samples $r_{1}^{\prime }(k),\ldots ,r_{n}^{\prime }(k)$ follow a normal distribution, so in fact an increased number of tied ranks does not affect the permutation strategy.

Hedrick’s (1987) [26] $D_{im}^{\prime }$ ranged from [0,1] was used in our proposed method. The reason was due to that using Hedrick’s (1987) $D_{im}^{\prime }$ makes cross-locus and cross-population comparisons uncomplicated [27]. We also carried out a simulation study assuming one causal SNP and 100 non-causal SNPs in a region under a scenario that NTR had lowest power in this study. The results showed that NTR had a bit higher power due to its consideration for linkage disequilibrium although all methods performed poorly. As suggested previously, the causal SNP that are in LD are more likely to end up together (segregate together) in a person (compared to independent alleles), thus LD would affect prevalence and the risk distribution in the population [48]. According to Morris et al. [49], an analysis based on haplotypes might be favorable over an analysis based on individual SNPs when multiple susceptibility alleles exist, particularly when linkage disequilibria between SNPs is not so strong [49].

The computation time for NTR, however, grows exponentially as the number of markers in a genomic region increases. This is a limitation for considering pairwise LD of all markers. When using our Linux-based workstation (Intel Xeon X5690 3.47-GHz CPU) to calculate the association for rare variants, the computation time was ~1 min for 50 SNPs in a genomic region, 6 min for 100 SNPs, and 43 min for 200 SNPs. However, SKAT-O required ~11 and ~21 s for 100 and 200 SNPs, respectively, in a genomic region. Despite the longer computation time and slightly higher false-positive rate, NTR might identify a greater number of genuine rare variants that are associated with complex diseases.

Our re-analysis of the DN dataset not only confirmed a landmark finding in genetic association studies but also discovered some potentially new candidate genes related to the disease. We caution, however, that the sample size in the DN dataset is relatively small, and hence these candidate genes require further investigation. Our research illustrates the important role of rare variants in DN and demonstrates that NTR is useful for analyzing real data. We conclude that different rare-variant association methods should complement each other toward the goal of dissecting possible risk factors for complex diseases.

## Supporting information

S1 Table Summary of results for eight mitochondrial genes from the SNP analysis. (PDF)

S2 Table Summary of results for 102 DN-related genes from the rare variant analysis. (PDF)

S3 Table The power for one causal rare SNP and 100 non-causal SNPs scenario. (PDF)

S4 Table The type I error rates under different scenarios. (PDF)
