# Supplementary material for: A non-threshold region-specific method for detecting rare variants in complex diseases
Source: PLoS One. 2017 Nov 30;12(11):e0188566. doi: 10.1371/journal.pone.0188566 (PMC5708778; doi:10.1371/journal.pone.0188566)
Supplement: S1 Table — (PDF) [file pone.0188566.s001.pdf]

Supplementary Table S1. Summary of results for eight mitochondrial genes from the SNP analysis.

Chr.	SNP	bp	Minor allele	Major allele	MAF	CASE (minor allele)	CASE (MAF)	Control (minor allele)	Control (MAF)	Trend test (p-value)	Gene symbol
6	rs1408705	163493288	G	A	0.07814	G	0.09478	G	0.06202	0.00024	*PACRG*
6	rs1588787	163274969	A	G	0.4365	A	0.4641	A	0.4125	0.00132	*PACRG*
22	rs5992495	18262984	G	T	0.1607	G	0.178	G	0.1445	0.00303	*TXNRD2*
6	rs4593343	163296767	A	G	0.4286	A	0.4538	A	0.4069	0.00317	*PACRG*
6	rs9347707	163281186	C	A	0.3552	C	0.3803	C	0.335	0.00318	*PACRG*
14	rs10151712	77122562	G	A	0.4783	G	0.5018	G	0.4629	0.00695	*SPTLC2*
14	rs12884431	77126958	A	G	0.1905	A	0.2086	A	0.1755	0.00815	*SPTLC2*
14	rs2072672	77057711	C	A	0.2519	C	0.2324	C	0.2708	0.00971	*SPTLC2*
6	rs13209731	163475722	C	T	0.1583	C	0.1403	C	0.1728	0.01001	*PACRG*
6	rs4329088	163315053	A	G	0.4991	A	0.4781	A	0.5172	0.01047	*PACRG*
14	rs10135955	77119487	T	C	0.4089	T	0.387	T	0.4241	0.01049	*SPTLC2*
6	rs2763994	163523460	A	G	0.2553	A	0.2351	A	0.2708	0.01053	*PACRG*
14	rs11621931	77110927	T	G	0.09636	T	0.08262	T	0.1074	0.01104	*SPTLC2*
8	rs2272636	144471261	G	T	0.452	G	0.4298	G	0.4751	0.01105	*TOP1MT*
6	rs742333	163639609	T	C	0.4368	T	0.4593	T	0.4147	0.01142	*PACRG*
8	rs724037	144473221	T	G	0.4521	T	0.4301	T	0.475	0.01172	*TOP1MT*
6	rs6907521	163531783	C	T	0.1437	C	0.1294	C	0.155	0.01519	*PACRG*
6	rs2151652	163260357	G	A	0.1951	G	0.212	G	0.1783	0.01531	*PACRG*
6	rs2763986	163512225	A	C	0.4307	A	0.4496	A	0.4136	0.01647	*PACRG*
6	rs6936963	163211290	T	C	0.07496	T	0.08505	T	0.06596	0.01689	*PACRG*
22	rs11541479	18286511	A	G	0.1466	A	0.1598	A	0.1346	0.01775	*TXNRD2*
12	rs3847971	119379672	C	G	0.3293	C	0.3499	C	0.3134	0.01955	*GATC*
14	rs2299922	77135153	C	T	0.4311	C	0.4124	C	0.4456	0.02461	*SPTLC2*
12	rs11065147	119439961	A	C	0.3267	A	0.3466	A	0.3114	0.02531	*COQ5*
6	rs9365506	163259322	A	G	0.4924	A	0.5104	A	0.474	0.02632	*PACRG*

12	rs7957424	119378701	G	A	0.4388	G	0.458	G	0.4218	0.02918	*GATC*
14	rs10129280	77138246	G	T	0.4327	G	0.4514	G	0.4207	0.03022	*SPTLC2*
12	rs8903	119362913	T	C	0.3297	T	0.3487	T	0.3152	0.03233	*COX6A1*
14	rs2299921	77134732	G	A	0.4306	G	0.4489	G	0.4186	0.03486	*SPTLC2*
14	rs2364603	77129784	C	T	0.07902	C	0.06926	C	0.08749	0.03665	*SPTLC2*
6	rs7742985	163497168	C	T	0.218	C	0.2011	C	0.2309	0.04015	*PACRG*
6	rs2143071	163551985	G	T	0.3455	G	0.3287	G	0.3627	0.04016	*PACRG*
14	rs17824567	77046203	G	T	0.1058	G	0.09538	G	0.1153	0.04016	*SPTLC2*
14	rs2299927	77136414	A	G	0.2598	A	0.2442	A	0.2711	0.04199	*SPTLC2*
8	SNP8-144478080	144478080	T	C	0.2885	T	0.2707	T	0.3042	0.04454	*TOP1MT*
6	rs9458662	163221034	G	A	0.455	G	0.4732	G	0.4412	0.0485	*PACRG*
6	rs13211224	163241222	G	A	0.07848	G	0.08688	G	0.07206	0.04865	*PACRG*
6	rs11964879	163447849	G	A	0.09289	G	0.08323	G	0.1014	0.0487	*PACRG*
6	rs10945884	163581992	C	T	0.4985	C	0.5146	C	0.4822	0.0511	*PACRG*
17	rs7214862	13941530	G	T	0.161	G	0.1476	G	0.1722	0.0524	*COX10*
6	rs13196605	163209026	G	T	0.08946	G	0.09781	G	0.08222	0.0603	*PACRG*
6	rs2476467	163466489	T	C	0.2171	T	0.2029	T	0.2292	0.06223	*PACRG*
6	rs6906182	163555870	A	G	0.3231	A	0.3086	A	0.3383	0.06869	*PACRG*
6	rs1041632	163139881	C	T	0.4407	C	0.4216	C	0.454	0.06931	*PACRG*
6	rs6909464	163321757	G	T	0.3532	G	0.3386	G	0.366	0.07122	*PACRG*
17	rs8076787	13913680	T	C	0.06316	T	0.05468	T	0.07087	0.07211	*COX10*
14	rs11159273	77079605	C	T	0.2897	C	0.2746	C	0.304	0.07226	*SPTLC2*
17	rs3785686	13945992	G	A	0.05523	G	0.06258	G	0.04817	0.07228	*COX10*
14	rs11159268	77043934	C	A	0.3239	C	0.3096	C	0.3355	0.07357	*SPTLC2*
17	rs2072278	13921375	G	T	0.0632	G	0.05474	G	0.07087	0.07357	*COX10*
6	rs10945886	163629800	A	C	0.2036	A	0.2177	A	0.1892	0.07453	*PACRG*
6	rs6455889	163639867	C	T	0.3387	C	0.3232	C	0.3516	0.0756	*PACRG*
6	rs2235994	163510957	C	T	0.4371	C	0.4239	C	0.4508	0.07671	*PACRG*

6	rs9306	163656194	C	T	0.4365	C	0.4216	C	0.4485	0.08092	*PACRG*
6	rs868708	163179250	A	G	0.3578	A	0.3408	A	0.3688	0.08386	*PACRG*
6	rs9347717	163370259	G	A	0.2795	G	0.2661	G	0.2894	0.08404	*PACRG*
17	rs34342426	13920783	T	A	0.06345	T	0.05529	T	0.07087	0.08505	*COX10*
6	rs16894545	163473738	T	C	0.1238	T	0.113	T	0.1321	0.08719	*PACRG*
14	rs2364602	77098556	A	G	0.07051	A	0.06318	A	0.07752	0.09165	*SPTLC2*
6	rs9346951	163495012	T	C	0.1254	T	0.1154	T	0.1334	0.09227	*PACRG*
6	rs10455921	163631667	C	A	0.3068	C	0.2917	C	0.3206	0.09392	*PACRG*
17	rs11078225	13976452	A	G	0.1938	A	0.1819	A	0.2033	0.09543	*COX10*
6	rs11964522	163583227	G	A	0.3177	G	0.3315	G	0.307	0.09865	*PACRG*
6	rs1536034	163379785	T	C	0.2798	T	0.267	T	0.289	0.1003	*PACRG*
8	rs3829018	144478002	A	G	0.105	A	0.1127	A	0.09622	0.1019	*TOP1MT*
6	rs3939989	163375772	G	A	0.4233	G	0.4083	G	0.433	0.1039	*PACRG*
6	rs13195963	163494881	A	G	0.1196	A	0.1302	A	0.1113	0.1062	*PACRG*
14	rs7145386	77062813	C	T	0.1189	C	0.1288	C	0.11	0.1071	*SPTLC2*
22	rs4646310	18308806	A	G	0.1989	A	0.1871	A	0.2082	0.1081	*TXNRD2*
6	rs742956	163520215	A	C	0.4774	A	0.4897	A	0.4679	0.1086	*PACRG*
6	rs10945874	163496012	T	C	0.1257	T	0.1162	T	0.1332	0.1115	*PACRG*
6	rs9458657	163197157	T	C	0.3698	T	0.3835	T	0.359	0.113	*PACRG*
17	rs3785688	13945617	T	C	0.1846	T	0.1965	T	0.1744	0.113	*COX10*
8	rs2293925	144463743	A	G	0.4433	A	0.458	A	0.4286	0.1179	*TOP1MT*
6	rs6936045	163377489	C	T	0.4233	C	0.4088	C	0.4324	0.119	*PACRG*
6	rs761621	163517526	A	C	0.1163	A	0.1075	A	0.1224	0.1213	*PACRG*
22	rs9617850	18298231	A	G	0.1984	A	0.1871	A	0.2073	0.1224	*TXNRD2*
6	rs12665063	163194296	G	A	0.05934	G	0.05225	G	0.06423	0.1273	*PACRG*
6	rs6916146	163630155	C	A	0.1853	C	0.1752	C	0.1945	0.1322	*PACRG*
6	rs12201059	163582619	A	C	0.04086	A	0.03524	A	0.04601	0.1343	*PACRG*
22	rs6518591	18304021	G	A	0.1977	G	0.1867	G	0.2063	0.1345	*TXNRD2*
6	rs6455864	163466626	T	C	0.2168	T	0.2047	T	0.2265	0.1349	*PACRG*

17	rs10521258	13954919	C	T	0.1252	C	0.1356	C	0.1157	0.137	*COX10*
6	rs7738930	163240115	T	C	0.01616	T	0.01276	T	0.01883	0.1455	*PACRG*
22	rs5748470	18287118	G	A	0.4926	G	0.4805	G	0.5044	0.1481	*TXNRD2*
22	rs2073750	18253357	T	C	0.2205	T	0.2305	T	0.2132	0.1482	*TXNRD2*
6	rs9346946	163348396	G	A	0.3671	G	0.3552	G	0.3769	0.1497	*PACRG*
6	rs1324965	163349000	A	C	0.3647	A	0.353	A	0.3746	0.15	*PACRG*
14	rs11629330	77092550	A	G	0.4803	A	0.4933	A	0.4684	0.1511	*SPTLC2*
22	rs1015471	18258514	A	G	0.3052	A	0.2951	A	0.3145	0.1534	*TXNRD2*
17	rs7213412	13955236	G	C	0.343	G	0.3303	G	0.3533	0.1549	*COX10*
22	rs3788317	18269825	T	G	0.2275	T	0.2372	T	0.2204	0.1574	*TXNRD2*
17	rs3785680	13993522	A	G	0.1196	A	0.1294	A	0.1109	0.1589	*COX10*
6	rs6922089	163623856	A	G	0.3048	A	0.292	A	0.3167	0.1605	*PACRG*
6	rs2985668	163520757	G	A	0.4724	G	0.4829	G	0.464	0.1613	*PACRG*
17	rs9903314	13941800	T	G	0.1851	T	0.1956	T	0.1761	0.1628	*COX10*
6	rs7760425	163209057	C	T	0.4171	C	0.4055	C	0.4265	0.1653	*PACRG*
6	rs4709670	163340616	A	G	0.3845	A	0.373	A	0.3937	0.1696	*PACRG*
6	rs12197200	163503111	A	G	0.136	A	0.1446	A	0.1318	0.1772	*PACRG*
6	rs9456839	163535952	C	T	0.1128	C	0.1191	C	0.1058	0.1784	*PACRG*
6	rs12210929	163567820	C	A	0.4659	C	0.455	C	0.479	0.1803	*PACRG*
14	rs17105922	77074102	G	A	0.1181	G	0.1264	G	0.1107	0.1808	*SPTLC2*
14	rs17105954	77081747	A	G	0.1181	A	0.1264	A	0.1107	0.1808	*SPTLC2*
22	rs1005873	18280075	G	A	0.1139	G	0.1071	G	0.1221	0.1838	*TXNRD2*
6	rs2025546	163350426	G	A	0.4968	G	0.5091	G	0.4878	0.1868	*PACRG*
6	rs6932546	163499576	A	G	0.1496	A	0.1582	A	0.1451	0.1877	*PACRG*
6	rs7768540	163633273	A	G	0.04671	A	0.05164	A	0.04208	0.1907	*PACRG*
22	rs756661	18285802	G	A	0.44	G	0.4288	G	0.4496	0.2012	*TXNRD2*
17	rs13183	14052333	T	C	0.3302	T	0.319	T	0.3422	0.2044	*COX10*
6	rs9456814	163193183	G	A	0.3869	G	0.3736	G	0.3953	0.2048	*PACRG*
22	rs1139793	18248218	A	G	0.2751	A	0.2837	A	0.2661	0.2145	*TXNRD2*

22	rs3788306	18252009	C	T	0.316	C	0.3074	C	0.3237	0.2289	*TXNRD2*
6	rs12213157	163560479	G	A	0.2004	G	0.2084	G	0.1938	0.2374	*PACRG*
22	rs4597638	18282526	C	T	0.06022	C	0.05529	C	0.06534	0.2381	*TXNRD2*
6	rs1399222	163248585	G	A	0.06908	G	0.07351	G	0.06596	0.2408	*PACRG*
22	rs2239893	18250831	T	C	0.3148	T	0.3066	T	0.3217	0.2444	*TXNRD2*
6	rs6922754	163571273	A	G	0.4741	A	0.4648	A	0.4856	0.2465	*PACRG*
17	rs9901800	13945320	G	T	0.1572	G	0.1659	G	0.1501	0.2527	*COX10*
17	rs10521257	13943140	G	A	0.3519	G	0.342	G	0.3616	0.2536	*COX10*
6	rs9458747	163542107	G	A	0.09577	G	0.0887	G	0.1008	0.2547	*PACRG*
17	rs10521264	14046599	T	C	0.05618	T	0.06083	T	0.05155	0.2553	*COX10*
6	rs9364679	163236402	G	A	0.3219	G	0.3127	G	0.3289	0.2558	*PACRG*
6	rs9458774	163625698	T	C	0.3443	T	0.3335	T	0.3549	0.2575	*PACRG*
6	rs12197662	163497911	T	G	0.3258	T	0.3147	T	0.3372	0.2607	*PACRG*
6	rs570437	163414285	T	G	0.3656	T	0.3727	T	0.3555	0.2652	*PACRG*
6	rs10945875	163498656	G	A	0.1507	G	0.158	G	0.1473	0.2652	*PACRG*
6	rs10945881	163551791	T	C	0.309	T	0.3177	T	0.2979	0.2679	*PACRG*
6	rs7759538	163530516	C	T	0.1144	C	0.1069	C	0.1199	0.2749	*PACRG*
17	rs3826368	13965942	T	C	0.1442	T	0.1507	T	0.1384	0.277	*COX10*
6	rs7750165	163617429	G	A	0.4389	G	0.4502	G	0.4275	0.284	*PACRG*
6	rs1877771	163238470	A	G	0.4727	A	0.4635	A	0.479	0.2932	*PACRG*
14	rs6574383	77094679	A	G	0.1667	A	0.1598	A	0.1713	0.2994	*SPTLC2*
6	rs908018	163239098	A	G	0.4724	A	0.4635	A	0.4784	0.308	*PACRG*
17	rs2302107	13913856	C	T	0.2691	C	0.2618	C	0.2785	0.3118	*COX10*
8	rs3812436	144483134	T	C	0.4405	T	0.4316	T	0.4514	0.3137	*TOP1MT*
6	rs520929	163473303	A	G	0.1707	A	0.178	A	0.1645	0.3145	*PACRG*
6	rs10455922	163643535	A	G	0.1931	A	0.1859	A	0.2012	0.3165	*PACRG*
6	rs7743292	163653452	A	G	0.07878	A	0.08394	A	0.0742	0.3165	*PACRG*
6	rs2874453	163229786	T	C	0.2618	T	0.2685	T	0.2542	0.3198	*PACRG*
17	rs2323096	14000752	T	C	0.3264	T	0.3177	T	0.3367	0.3246	*COX10*

22	rs3788305	18251778	G	A	0.4615	G	0.4538	G	0.4707	0.3274	*TXNRD2*
22	rs1044732	18243142	C	T	0.1292	C	0.1348	C	0.1262	0.329	*TXNRD2*
17	rs12943936	13978196	A	G	0.4223	A	0.4134	A	0.434	0.3317	*COX10*
6	rs536980	163463689	C	T	0.2831	C	0.2895	C	0.2769	0.334	*PACRG*
22	rs16984299	18243593	C	T	0.1337	C	0.1391	C	0.1307	0.3382	*TXNRD2*
22	rs7288170	18244252	G	C	0.1332	G	0.1387	G	0.1308	0.3404	*TXNRD2*
6	rs576381	163448652	A	G	0.2939	A	0.3007	A	0.2855	0.3425	*PACRG*
17	rs1017077	13945797	T	G	0.1514	T	0.1588	T	0.1456	0.3454	*COX10*
14	rs4903606	77106688	A	G	0.3563	A	0.3633	A	0.3503	0.3488	*SPTLC2*
22	rs7288061	18244239	T	C	0.129	T	0.1343	T	0.1262	0.3503	*TXNRD2*
6	rs568619	163450415	T	C	0.2909	T	0.2976	T	0.2825	0.3507	*PACRG*
6	rs9356065	163159109	G	A	0.06375	G	0.05893	G	0.067	0.3548	*PACRG*
22	rs2020917	18308884	T	C	0.2838	T	0.2892	T	0.2785	0.3554	*TXNRD2*
6	rs2763983	163409658	T	C	0.2174	T	0.2236	T	0.2099	0.3593	*PACRG*
8	rs2467894	144483142	A	C	0.4968	A	0.5043	A	0.4873	0.3618	*TOP1MT*
22	rs9605031	18301378	T	C	0.2842	T	0.2895	T	0.2791	0.3626	*TXNRD2*
6	rs7759501	163121369	T	G	0.06614	T	0.06204	T	0.06866	0.3654	*PACRG*
6	rs2235993	163510246	T	C	0.4013	T	0.3961	T	0.4081	0.3709	*PACRG*
6	rs6903758	163656492	G	A	0.3589	G	0.3522	G	0.3654	0.3739	*PACRG*
22	rs1800706	18308022	A	G	0.2801	A	0.2852	A	0.2752	0.3749	*TXNRD2*
6	rs524763	163450497	T	C	0.2952	T	0.3017	T	0.2872	0.3836	*PACRG*
6	rs9346937	163199969	A	G	0.4292	A	0.4221	A	0.4341	0.3885	*PACRG*
6	rs504844	163471366	G	A	0.1868	G	0.1932	G	0.1822	0.3948	*PACRG*
22	rs8140265	18301641	A	G	0.2844	A	0.2892	A	0.2796	0.3957	*TXNRD2*
6	rs9364672	163151074	C	T	0.3083	C	0.2993	C	0.3145	0.4	*PACRG*
6	rs2747688	163496283	C	T	0.3252	C	0.3196	C	0.3306	0.4017	*PACRG*
12	rs503335	119444440	T	C	0.07139	T	0.06744	T	0.07475	0.4101	*COQ5*
6	rs10945890	163649996	C	T	0.2491	C	0.2564	C	0.2442	0.4102	*PACRG*
6	rs7760002	163603512	T	C	0.3798	T	0.3888	T	0.3704	0.415	*PACRG*

17	rs1029688	13971200	C	T	0.03588	C	0.03281	C	0.03774	0.4182	*COX10*
6	rs1575472	163416279	G	A	0.1307	G	0.1245	G	0.1346	0.4244	*PACRG*
6	rs1828819	163253575	G	A	0.2434	G	0.2363	G	0.2472	0.4263	*PACRG*
6	rs4709683	163562195	G	A	0.3208	G	0.3269	G	0.3117	0.4285	*PACRG*
22	rs1978058	18288219	T	C	0.3884	T	0.3821	T	0.3953	0.4309	*TXNRD2*
6	rs6927758	163639105	T	C	0.4345	T	0.4276	T	0.4424	0.4325	*PACRG*
17	rs1802618	14052012	C	T	0.1425	C	0.147	C	0.1384	0.4336	*COX10*
22	rs13054371	18247189	C	T	0.4665	C	0.4611	C	0.474	0.435	*TXNRD2*
14	rs17751562	77047246	G	A	0.09818	G	0.09478	G	0.1014	0.436	*SPTLC2*
6	rs12529659	163652929	C	T	0.09783	C	0.1021	C	0.09524	0.4374	*PACRG*
6	rs520273	163456597	T	G	0.3531	T	0.3584	T	0.3494	0.4605	*PACRG*
22	rs3788310	18260340	A	G	0.09289	A	0.08942	A	0.0969	0.4634	*TXNRD2*
6	rs1122682	163543967	A	G	0.3043	A	0.3098	A	0.2973	0.4736	*PACRG*
6	rs13217038	163529683	A	G	0.1124	A	0.1071	A	0.1159	0.4744	*PACRG*
6	rs2747690	163503276	T	G	0.1601	T	0.1628	T	0.1545	0.4773	*PACRG*
14	rs1554957	77077624	A	G	0.284	A	0.2895	A	0.2796	0.4784	*SPTLC2*
6	rs547434	163483664	G	A	0.3352	G	0.3293	G	0.34	0.4893	*PACRG*
6	rs9456841	163540606	T	C	0.3178	T	0.323	T	0.3112	0.4899	*PACRG*
17	rs16949080	13992260	A	G	0.1002	A	0.1039	A	0.09801	0.4977	*COX10*
17	rs2323090	13914158	G	T	0.2037	G	0.208	G	0.1982	0.5039	*COX10*
12	rs2235217	119368711	T	C	0.3443	T	0.3374	T	0.3492	0.5053	*GATC*
6	rs1399221	163204028	G	A	0.4426	G	0.4377	G	0.4463	0.5061	*PACRG*
6	rs4709696	163642258	T	C	0.1412	T	0.1472	T	0.1386	0.5241	*PACRG*
6	rs9456815	163193316	T	C	0.169	T	0.1636	T	0.1722	0.5254	*PACRG*
6	rs1022611	163542648	A	G	0.316	A	0.3207	A	0.3099	0.5279	*PACRG*
6	rs2874514	163467193	G	T	0.3619	G	0.3688	G	0.3583	0.5285	*PACRG*
6	rs11759691	163646219	T	G	0.121	T	0.1258	T	0.119	0.5321	*PACRG*
6	rs2476468	163467043	A	G	0.3466	A	0.3406	A	0.3511	0.535	*PACRG*
6	rs11968371	163211943	G	A	0.2337	G	0.2394	G	0.2317	0.5372	*PACRG*

6	rs949902	163170768	A	C	0.1055	A	0.1083	A	0.103	0.5389	*PACRG*
22	rs9605030	18301137	T	C	0.1394	T	0.1423	T	0.1364	0.5429	*TXNRD2*
6	rs498657	163471982	C	T	0.1965	C	0.192	C	0.1993	0.5468	*PACRG*
6	rs483222	163475147	G	A	0.1669	G	0.1707	G	0.1628	0.5511	*PACRG*
6	rs1205665	163488065	C	T	0.3626	C	0.3672	C	0.3575	0.5646	*PACRG*
14	rs11159269	77046078	T	C	0.171	T	0.1671	T	0.1728	0.5663	*SPTLC2*
14	rs17824646	77076438	T	C	0.09518	T	0.09235	T	0.09745	0.5669	*SPTLC2*
6	rs9346938	163206342	A	G	0.2728	A	0.267	A	0.2757	0.5671	*PACRG*
6	rs2747691	163504963	T	C	0.2783	T	0.2713	T	0.2805	0.5688	*PACRG*
6	rs9458750	163543693	C	A	0.3196	C	0.3238	C	0.3134	0.5709	*PACRG*
17	rs11078234	14051626	G	A	0.1424	G	0.1456	G	0.1397	0.5789	*COX10*
14	rs1063271	77044743	C	T	0.171	C	0.1673	C	0.1726	0.5864	*SPTLC2*
14	rs1063270	77044856	T	G	0.171	T	0.1673	T	0.1726	0.5864	*SPTLC2*
12	rs10849757	119448397	A	G	0.3467	A	0.3408	A	0.3505	0.5955	*COQ5*
14	rs2284230	77085650	T	C	0.2105	T	0.2145	T	0.2057	0.5957	*SPTLC2*
6	rs6917499	163168579	C	T	0.1495	C	0.1458	C	0.1506	0.5984	*PACRG*
6	rs7770851	163623098	T	G	0.3849	T	0.3913	T	0.3778	0.6019	*PACRG*
12	rs11065145	119431802	G	A	0.3466	G	0.3408	G	0.3503	0.6029	*COQ5*
17	rs11870213	13918187	A	G	0.4186	A	0.4247	A	0.4153	0.6037	*COX10*
6	rs4470828	163610419	C	T	0.3892	C	0.3955	C	0.3821	0.6051	*PACRG*
6	rs10455920	163489262	C	T	0.1466	C	0.1495	C	0.1467	0.606	*PACRG*
12	rs7471	119425598	C	T	0.3469	C	0.3412	C	0.3505	0.6137	*COQ5*
12	rs10774549	119375555	T	C	0.3458	T	0.3402	T	0.3494	0.6168	*GATC*
17	rs2323091	13967540	C	T	0.2835	C	0.2868	C	0.2807	0.6179	*COX10*
6	rs16888819	163558257	T	C	0.1395	T	0.1428	T	0.1379	0.6326	*PACRG*
8	rs2450775	144488276	C	A	0.09187	C	0.09329	C	0.09035	0.6342	*TOP1MT*
6	rs12526892	163641063	T	C	0.3002	T	0.305	T	0.2979	0.6437	*PACRG*
6	rs13202719	163630381	G	A	0.2587	G	0.2618	G	0.2572	0.6443	*PACRG*
12	rs17431446	119368674	T	C	0.05905	T	0.05772	T	0.06035	0.647	*GATC*

14	rs10147810	77060859	T	C	0.3616	T	0.3579	T	0.3647	0.6526	*SPTLC2*
6	rs9458711	163347558	A	G	0.01681	A	0.01766	A	0.01559	0.6536	*PACRG*
17	rs9909412	13963840	C	A	0.4818	C	0.4763	C	0.485	0.6594	*COX10*
14	rs11846597	77059691	G	A	0.1914	G	0.187	G	0.1948	0.6703	*SPTLC2*
6	rs13212746	163123082	C	T	0.2106	C	0.2078	C	0.2143	0.6758	*PACRG*
6	rs11753570	163650662	T	G	0.1119	T	0.1136	T	0.1091	0.6767	*PACRG*
6	rs12529332	163534710	C	A	0.2777	C	0.2821	C	0.2746	0.6806	*PACRG*
6	rs10945865	163252841	T	C	0.2218	T	0.2181	T	0.2226	0.6811	*PACRG*
6	rs9347712	163291048	G	T	0.0824	G	0.08354	G	0.07971	0.6826	*PACRG*
6	rs520741	163427477	G	A	0.2444	G	0.2473	G	0.2403	0.6862	*PACRG*
6	rs4458655	163141782	C	T	0.1351	C	0.1324	C	0.1357	0.6873	*PACRG*
6	rs9347691	163169635	G	A	0.05647	G	0.0542	G	0.05759	0.6912	*PACRG*
6	rs9365548	163653428	C	T	0.3291	C	0.3329	C	0.328	0.6995	*PACRG*
6	rs548705	163441358	A	G	0.2685	A	0.271	A	0.2647	0.7042	*PACRG*
22	rs3788314	18269423	A	G	0.4726	A	0.475	A	0.4701	0.7084	*TXNRD2*
6	rs4708987	163643906	T	C	0.3366	T	0.3352	T	0.3422	0.7088	*PACRG*
22	rs1139795	18247771	T	C	0.1459	T	0.1478	T	0.1463	0.7119	*TXNRD2*
14	rs12433198	77112060	A	G	0.1126	A	0.1106	A	0.1138	0.714	*SPTLC2*
17	rs6502336	13948585	C	T	0.47	C	0.466	C	0.4729	0.7146	*COX10*
6	rs12214680	163645834	A	G	0.09959	A	0.09842	A	0.1024	0.7154	*PACRG*
6	rs233	163623998	G	A	0.2576	G	0.26	G	0.2564	0.7215	*PACRG*
6	rs556085	163442156	G	A	0.3915	G	0.3879	G	0.392	0.728	*PACRG*
6	rs573070	163427041	T	C	0.2443	T	0.2467	T	0.2406	0.7308	*PACRG*
6	rs485964	163392393	G	A	0.3184	G	0.3159	G	0.3189	0.7414	*PACRG*
6	rs561440	163406927	G	A	0.3196	G	0.3171	G	0.32	0.7468	*PACRG*
17	rs2856177	14041754	G	A	0.4107	G	0.4089	G	0.4097	0.7473	*COX10*
6	rs4709679	163506559	A	C	0.2186	A	0.2199	A	0.2215	0.7506	*PACRG*
6	rs3799339	163646416	G	A	0.2429	G	0.2482	G	0.2428	0.7519	*PACRG*
22	rs8141691	18289426	A	G	0.3769	A	0.3809	A	0.3743	0.7526	*TXNRD2*

22	rs8141610	18250147	T	C	0.1412	T	0.1428	T	0.1415	0.7551	*TXNRD2*
6	rs1514338	163152322	C	T	0.2444	C	0.2406	C	0.247	0.7552	*PACRG*
6	rs535479	163449066	C	T	0.3876	C	0.3844	C	0.388	0.7569	*PACRG*
6	rs12211969	163653158	G	A	0.1889	G	0.1871	G	0.191	0.7577	*PACRG*
17	rs8075612	14005372	T	G	0.3727	T	0.3767	T	0.367	0.7636	*COX10*
22	rs5748469	18287099	A	C	0.3601	A	0.3585	A	0.3614	0.7654	*TXNRD2*
6	rs1555064	163597017	G	A	0.3001	G	0.2968	G	0.3045	0.7668	*PACRG*
14	rs12435993	77133193	C	A	0.2381	C	0.2363	C	0.2428	0.7726	*SPTLC2*
22	rs5748485	18303415	A	G	0.421	A	0.425	A	0.4179	0.7739	*TXNRD2*
6	rs2294458	163586326	G	T	0.1096	G	0.11	G	0.1074	0.7748	*PACRG*
6	rs9295206	163246682	G	A	0.3465	G	0.3421	G	0.3474	0.7771	*PACRG*
6	rs9458753	163549777	A	G	0.3404	A	0.342	A	0.3365	0.7793	*PACRG*
6	rs10945861	163093668	T	C	0.4126	T	0.4106	T	0.4163	0.7808	*PACRG*
6	rs577398	163448796	A	G	0.3879	A	0.385	A	0.388	0.7857	*PACRG*
6	rs9458689	163307367	T	C	0.07315	T	0.07351	T	0.07143	0.7992	*PACRG*
6	rs9458749	163543609	T	G	0.01028	T	0.009721	T	0.01052	0.8005	*PACRG*
6	rs13195186	163079177	G	A	0.3719	G	0.3694	G	0.3726	0.8029	*PACRG*
22	rs1012157	18289977	A	G	0.2115	A	0.2122	A	0.2103	0.805	*TXNRD2*
6	rs6908925	163581503	G	A	0.1789	G	0.1792	G	0.1755	0.8053	*PACRG*
6	rs13209100	163079340	A	G	0.1052	A	0.1039	A	0.1052	0.8054	*PACRG*
17	rs1050223	14052640	T	C	0.3433	T	0.3425	T	0.3416	0.8139	*COX10*
17	rs12449610	13998813	C	T	0.3733	C	0.3767	C	0.3681	0.8164	*COX10*
6	rs9356058	163071389	C	T	0.4077	C	0.4101	C	0.4063	0.8187	*PACRG*
6	rs6908157	163464695	T	C	0.1019	T	0.1009	T	0.103	0.8229	*PACRG*
6	rs547694	163396784	G	A	0.4643	G	0.4597	G	0.4638	0.8236	*PACRG*
22	rs5993875	18295326	A	G	0.4207	A	0.4241	A	0.4181	0.8263	*TXNRD2*
22	rs5746847	18301003	T	C	0.4201	T	0.4235	T	0.4175	0.8282	*TXNRD2*
17	rs2159132	13946164	G	A	0.4127	G	0.4156	G	0.4131	0.833	*COX10*
22	rs9306229	18292015	T	C	0.2235	T	0.2207	T	0.2261	0.8354	*TXNRD2*

6	rs9347686	163091960	C	T	0.4195	C	0.418	C	0.4225	0.8364	*PACRG*
6	rs11759099	163651905	T	C	0.1419	T	0.1416	T	0.1417	0.8417	*PACRG*
6	rs6455894	163653010	A	C	0.1764	A	0.1794	A	0.1761	0.8429	*PACRG*
17	rs8077302	13921075	G	A	0.495	G	0.4939	G	0.4978	0.8435	*COX10*
6	rs16894769	163651778	T	C	0.1119	T	0.113	T	0.113	0.8442	*PACRG*
17	rs8073382	13989492	A	G	0.373	A	0.3761	A	0.3681	0.8453	*COX10*
6	rs7764309	163082340	G	T	0.211	G	0.2098	G	0.2131	0.853	*PACRG*
6	rs12197625	163651492	A	G	0.09988	A	0.09964	A	0.1019	0.853	*PACRG*
6	rs6940541	163216869	A	C	0.0802	A	0.0808	A	0.07973	0.855	*PACRG*
12	rs3742049	119438873	T	C	0.1122	T	0.11	T	0.1118	0.8553	*COQ5*
6	rs9347700	163232680	T	C	0.0802	T	0.0808	T	0.07973	0.8556	*PACRG*
14	rs2272589	77093535	T	C	0.1987	T	0.1965	T	0.201	0.8584	*SPTLC2*
14	rs10147023	77045678	T	C	0.07227	T	0.0723	T	0.07032	0.8607	*SPTLC2*
6	rs915013	163244832	C	T	0.3369	C	0.3354	C	0.3359	0.8609	*PACRG*
6	rs9356075	163237264	A	C	0.1578	A	0.1567	A	0.1578	0.8643	*PACRG*
6	rs554427	163469861	G	A	0.2456	G	0.2454	G	0.2425	0.8644	*PACRG*
6	rs558017	163470259	C	A	0.2456	C	0.2454	C	0.2425	0.8644	*PACRG*
6	rs2276201	163069487	C	T	0.2741	C	0.2722	C	0.2719	0.8654	*PACRG*
6	rs1008295	163530086	C	T	0.2894	C	0.2898	C	0.2901	0.8719	*PACRG*
6	rs10806767	163132289	T	C	0.4224	T	0.4241	T	0.4219	0.8746	*PACRG*
6	rs11968630	163337489	T	C	0.01881	T	0.01823	T	0.01885	0.875	*PACRG*
6	rs763598	163545631	A	G	0.3783	A	0.3803	A	0.3769	0.8795	*PACRG*
6	rs9365505	163232835	G	A	0.4903	G	0.4884	G	0.4906	0.8806	*PACRG*
17	rs3826366	14048304	C	T	0.1986	C	0.1987	C	0.1971	0.8851	*COX10*
6	rs4709655	163200194	T	C	0.1119	T	0.1112	T	0.1118	0.8963	*PACRG*
6	rs11966414	163123202	T	C	0.1607	T	0.161	T	0.1578	0.897	*PACRG*
6	rs7744306	163096141	C	T	0.3137	C	0.3129	C	0.3139	0.8984	*PACRG*
6	rs9346956	163654194	T	C	0.4213	T	0.4198	T	0.4236	0.8989	*PACRG*
17	rs12452172	14049774	C	T	0.4577	C	0.4586	C	0.4607	0.9015	*COX10*

14	rs12589478	77060055	A	G	0.2574	A	0.2585	A	0.2567	0.9055	*SPTLC2*
6	rs13202023	163227726	C	T	0.07932	C	0.07959	C	0.07918	0.9115	*PACRG*
22	rs5993853	18259938	T	C	0.306	T	0.3054	T	0.3056	0.9124	*TXNRD2*
17	rs17678774	13988027	C	T	0.3252	C	0.325	C	0.3278	0.9161	*COX10*
6	rs511826	163482067	T	C	0.2889	T	0.2886	T	0.2888	0.9169	*PACRG*
6	rs7762226	163357726	C	T	0.1419	C	0.1409	C	0.1412	0.921	*PACRG*
6	rs4708990	163645488	T	C	0.4553	T	0.4563	T	0.4585	0.9301	*PACRG*
6	rs6455848	163073228	T	G	0.1677	T	0.1677	T	0.165	0.9335	*PACRG*
17	rs2529626	14045669	A	G	0.179	A	0.1788	A	0.1783	0.9346	*COX10*
6	rs11961035	163112028	A	G	0.1604	A	0.1604	A	0.1578	0.9355	*PACRG*
6	rs566126	163474031	G	A	0.3593	G	0.3584	G	0.3588	0.9382	*PACRG*
6	rs1333955	163133444	C	T	0.4718	C	0.4708	C	0.4695	0.9421	*PACRG*
6	rs6455854	163224280	G	A	0.4268	G	0.4282	G	0.4269	0.9444	*PACRG*
22	rs9332314	18308000	T	C	0.02293	T	0.02251	T	0.0227	0.9488	*TXNRD2*
8	rs2450772	144484791	A	G	0.1152	A	0.1142	A	0.1152	0.9549	*TOP1MT*
6	rs7738903	163240064	T	C	0.1945	T	0.1932	T	0.1932	0.9589	*PACRG*
6	rs9458781	163641174	G	A	0.4508	G	0.4494	G	0.4512	0.9589	*PACRG*
6	rs4709680	163520791	G	A	0.2723	G	0.2746	G	0.2724	0.9592	*PACRG*
6	rs13202482	163190133	T	C	0.08524	T	0.08516	T	0.08583	0.9621	*PACRG*
6	rs1205662	163486457	T	C	0.3216	T	0.3232	T	0.321	0.9692	*PACRG*
6	rs9347684	163071814	C	T	0.2029	C	0.2029	C	0.2037	0.9739	*PACRG*
6	rs9355403	163132568	A	G	0.2087	A	0.2086	A	0.2093	0.9769	*PACRG*
6	rs4709698	163642754	C	T	0.4553	C	0.4568	C	0.4579	0.9834	*PACRG*
6	rs13200025	163626203	T	C	0.4838	T	0.4842	T	0.485	0.9852	*PACRG*
6	rs1408710	163368740	C	T	0.1437	C	0.1422	C	0.1434	0.9897	*PACRG*
17	rs9901316	14041963	A	G	0.06816	A	0.06934	A	0.06755	0.9952	*COX10*
6	rs571644	163397094	G	A	0.4668	G	0.4654	G	0.4646	0.9975	*PACRG*
