# Supplementary material for: A non-threshold region-specific method for detecting rare variants in complex diseases
Source: PLoS One. 2017 Nov 30;12(11):e0188566. doi: 10.1371/journal.pone.0188566 (PMC5708778; doi:10.1371/journal.pone.0188566)
Supplement: S2 Table — (PDF) [file pone.0188566.s002.pdf]

S2 Table. Summary of results for 102 DN-related genes from the rare variant analysis.

Gene symbol	Chr[a]	Num[b]	NTR[c]	SKAT[c]	SKAT-O[c]	CMC[c]	WSS[c]
NR1H3	11	7	0.001333	0.028410	0.012010	0.098176	0.002703
CTSH	15	43	0.001795	0.263040	0.337420	0.978416	0.114007
GAS6	13	8	0.005530	0.249080	0.143320	0.018992	0.003916
CR1	1	17	0.011830	0.097130	0.027000	0.300985	0.003365
REN	1	8	0.013295	0.245090	0.164800	0.159001	0.021084
PPARG	3	119	0.016496	0.427690	0.591520	0.899883	0.931892
TEK	9	98	0.022544	0.869360	0.556250	0.245792	0.010016
CLEC3B	3	7	0.033660	0.351060	0.179370	0.520919	0.031352
GSS	20	17	0.040151	0.191450	0.268460	0.292601	0.931963
CD34	1	14	0.067732	0.581770	0.473390	0.088015	0.039521
COL4A4	2	62	0.077948	0.039690	0.009270	0.075902	0.014213
NOTCH4	6	2	0.091941	0.241610	0.241560	0.556648	0.121875
PTGER4	5	3	0.094654	0.647680	0.744930	0.253658	0.093249
PDE2A	11	29	0.096894	0.382250	0.284720	0.074661	0.094730
APOE	19	2	0.103743	0.187260	0.178400	0.528441	0.078635
TYRO3	15	8	0.106625	0.239300	0.239530	0.849723	0.549947
GRIK2	6	192	0.108717	0.060450	0.111890	0.000382	0.441434
CD36	7	86	0.119175	0.428260	0.589480	0.450156	0.101434
AQP1	7	13	0.119324	0.067590	0.060490	0.596026	0.072560
WNT6	2	2	0.131469	0.322660	0.290080	0.378828	0.094832
FABP1	2	3	0.140578	0.169100	0.122280	0.293341	0.102981
PPARD	6	63	0.142734	0.667290	0.808530	0.979034	0.117358
SPOCK1	5	157	0.145647	0.862780	0.818440	0.283399	0.114668
CHI3L1	1	15	0.151319	0.002370	0.002420	0.429640	0.128915
GAS2L1	22	4	0.159420	0.143000	0.079410	0.537215	0.091239
NOTCH4	6	62	0.165858	0.589130	0.559800	0.374442	0.752833
IFIT1	10	5	0.177880	0.602240	0.592760	0.667140	0.224947
SLC7A11	4	15	0.187487	0.321980	0.365700	0.800403	0.452807
FGF1	5	52	0.202092	0.101360	0.164460	0.096572	0.466536
WNT2B	1	17	0.214481	0.502800	0.469140	0.725076	0.774006
CCL3	17	2	0.217591	0.198450	0.198870	0.230836	0.222017
ACE	17	14	0.226251	0.141800	0.239030	0.612289	0.315098
CXADR	21	26	0.249635	0.019930	0.010870	0.554741	0.434994
SPP1	4	6	0.253507	0.507920	0.566650	0.405491	0.719129
F2R	5	6	0.261547	0.276120	0.260200	0.193927	0.093552

GPLD1	6	50	0.282878	0.888000	1.000000	0.101526	0.976852
AGT	1	9	0.291281	0.572200	0.608430	0.240573	0.329892
ADIPOQ	3	7	0.302653	0.016560	0.014870	0.133652	0.510036
PTGIS	20	25	0.316932	0.535850	0.419630	0.887951	0.443584
BMP4	14	13	0.330002	0.465180	0.477410	0.351268	0.373487
PPM1F	22	11	0.346092	0.193290	0.295060	0.648920	0.767987
CARS	11	13	0.373788	0.296660	0.401160	0.753812	0.081809
WNT4	1	10	0.378908	0.181960	0.161210	0.726593	0.508865
ACACA	17	49	0.385686	0.286800	0.286710	0.791805	0.510369
TNNT2	1	17	0.390031	0.348200	0.469650	0.459893	0.638757
BMP2	20	9	0.398781	0.522830	0.592070	0.689971	0.274477
MAGI2	7	545	0.400748	0.455820	0.665830	0.000000	0.459284
ELMO1	7	190	0.401444	0.516590	0.659460	0.335188	0.613678
PKLR	1	5	0.403626	0.721350	0.729370	0.825428	0.508427
HYAL1	3	2	0.413586	0.935760	0.817930	0.947641	0.477123
GLIPR2	9	10	0.420575	0.098980	0.101640	0.483554	0.386984
ANGPT4	20	44	0.425206	0.021940	0.030370	0.030614	0.695243
LPL	8	20	0.437312	0.701980	0.531870	0.610418	0.522721
STAT1	2	25	0.454969	0.341680	0.288710	0.649181	0.562182
CTNNB1	3	10	0.455986	0.240990	0.100640	0.394799	0.827086
AIF1	6	3	0.468547	0.345610	0.458870	0.669305	0.408977
LEF1	4	19	0.468788	0.376220	0.254580	0.220479	0.365172
ABCG1	21	89	0.470433	0.791680	0.493510	0.943713	0.776285
PLA2R1	2	46	0.488297	0.874780	1.000000	0.627898	0.512675
CRHBP	5	6	0.516033	0.451780	0.515790	0.909272	0.574550
SLC12A3	16	34	0.531483	0.817490	0.779200	0.904440	0.701141
IGFBP2	2	8	0.551625	0.304460	0.460280	0.739646	0.275971
AKR1C3	10	28	0.558513	0.077660	0.132460	0.005263	0.317403
ABCA1	9	169	0.566131	0.450020	0.496760	0.674011	0.461321
MYC	8	9	0.582698	0.688720	0.451120	0.798855	0.568983
GMDS	6	198	0.583613	0.085100	0.134350	0.323258	0.311386
INS	11	11	0.609289	0.461570	0.606370	0.532487	0.518415
CDS1	4	15	0.619194	0.853540	0.843330	0.914091	0.719690
TNFSF10	3	17	0.622026	0.936700	1.000000	0.470494	0.516090
AIF1	6	4	0.629597	1.000000	0.801890	0.976944	0.649271
MSR1	8	35	0.636577	0.553190	0.329330	0.737851	0.250358
ENPEP	4	18	0.637465	0.144240	0.202690	0.336483	0.873461
HBB	11	4	0.650750	0.516300	0.640560	0.557442	0.524837

TCF21	6	3	0.662970	1.000000	1.000000	0.854671	0.547540
FOXC1	6	2	0.671860	0.343500	0.351860	0.820170	0.419632
BRCA2	13	29	0.683308	0.750960	0.828390	0.658205	0.763916
CLU	8	7	0.689326	0.514510	0.570490	0.681759	0.275239
CAPN3	15	19	0.694872	0.455290	0.603750	0.970452	0.828459
LILRB1	19	2	0.696388	0.759160	0.688840	0.907227	0.638848
SERPINC1	1	6	0.705086	0.623160	0.752110	0.893271	0.603332
MLXIPL	7	8	0.724294	1.000000	1.000000	0.662243	0.624888
TUSC3	8	100	0.728887	0.185050	0.295070	0.086051	0.647484
SPG7	16	20	0.733175	0.650640	0.698530	0.704083	0.909657
WNT16	7	8	0.758896	0.526460	0.470440	0.947723	0.702972
UMOD	16	7	0.760562	0.618620	0.606600	0.852487	0.695890
ARHGEF15	17	8	0.762592	1.000000	1.000000	0.814528	0.759154
PTGDS	9	6	0.763009	0.395290	0.395760	0.841604	0.702881
APOD	3	5	0.765452	0.021210	0.010540	0.175092	0.383249
FRMD3	9	132	0.769746	0.713880	0.894640	0.598118	0.584670
F3	1	6	0.770555	0.683280	0.709670	0.742847	0.586707
SREBF1	17	7	0.774593	0.628580	0.628730	0.895553	0.457817
SCD	10	5	0.782778	0.174260	0.169390	0.626798	0.584215
PTPRM	18	237	0.808233	0.282260	0.448230	0.000528	0.300414
MYO1B	2	25	0.822119	0.386370	0.396090	0.965016	0.533473
FBP1	9	11	0.830613	0.661220	0.322450	0.798868	0.368266
XDH	2	59	0.846946	0.177740	0.268180	0.844039	0.991100
NEBL	10	138	0.893022	0.374830	0.541410	0.338132	0.554994
PPARA	22	53	0.931002	0.328720	0.507930	0.225940	0.895152
ACO1	9	22	0.931978	0.437960	0.283630	0.341116	0.652883
CDKN1A	6	4	0.933962	0.950390	1.000000	0.966421	0.666962
CCL16	17	4	0.966284	0.446830	0.509500	0.955058	0.727201
SERPIND1	22	8	0.995937	0.871790	0.836410	0.925287	0.871827

[a]Chromosome. [b]The number of SNPs located within the gene. [c]The p-values of the methods.
