# Supplementary material for: A non-threshold region-specific method for detecting rare variants in complex diseases
Source: PLoS One. 2017 Nov 30;12(11):e0188566. doi: 10.1371/journal.pone.0188566 (PMC5708778; doi:10.1371/journal.pone.0188566)
Supplement: S3 Table — (PDF) [file pone.0188566.s003.pdf]

S3 Table. The power for one causal rare SNP and 100 non-causal SNPs scenario.

Power	OR=1.2, ρ=0.2<br>(# of non-causal rare SNPs:100)
NTR	0.079
SKAT	0.057
SKAT-O	0.061
CMC	0.061
WSS	0.060
