# Supplementary material for: A non-threshold region-specific method for detecting rare variants in complex diseases
Source: PLoS One. 2017 Nov 30;12(11):e0188566. doi: 10.1371/journal.pone.0188566 (PMC5708778; doi:10.1371/journal.pone.0188566)
Supplement: S4 Table — (PDF) [file pone.0188566.s004.pdf]

S4 Table. The type I error rates under different scenarios.

Scenario	number of non-causal variants	NTR	SKAT-O	SKAT	CMC	WSS
Independent, Rare variants	0	0.045	0.049	0.063	0.042	0.041
	4	0.055	0.051	0.042	0.056	0.061
	8	0.05	0.056	0.047	0.056	0.068
	16	0.044	0.047	0.046	0.055	0.055
	32	0.052	0.050	0.042	0.044	0.060
Independent, Rare+Common variants	0	0.046	0.048	0.048	0.051	0.048
	4	0.059	0.062	0.054	0.058	0.064
	8	0.052	0.051	0.052	0.059	0.061
	16	0.049	0.046	0.046	0.057	0.050
	32	0.044	0.053	0.046	0.045	0.046
rho=2, Rare variants	0	0.045	0.052	0.051	0.050	0.047
	4	0.054	0.052	0.051	0.055	0.053
	8	0.048	0.051	0.043	0.050	0.050
	16	0.042	0.055	0.058	0.043	0.050
	32	0.055	0.041	0.044	0.059	0.043
rho=2, Rare+Common variants	0	0.045	0.055	0.056	0.040	0.048
	4	0.052	0.054	0.044	0.059	0.051
	8	0.041	0.052	0.055	0.051	0.042
	16	0.048	0.050	0.051	0.048	0.047
	32	0.057	0.047	0.047	0.056	0.054
rho=4, Rare variants	0	0.042	0.051	0.045	0.049	0.045
	4	0.055	0.060	0.053	0.050	0.047
	8	0.055	0.046	0.048	0.052	0.052
	16	0.051	0.061	0.047	0.046	0.044
	32	0.046	0.043	0.046	0.048	0.050
rho=4, Rare+Common variants	0	0.04	0.055	0.053	0.051	0.047
	4	0.044	0.055	0.065	0.052	0.043
	8	0.052	0.055	0.048	0.068	0.056
	16	0.052	0.053	0.048	0.058	0.049
	32	0.048	0.042	0.036	0.053	0.049
rho=6, Rare variants	0	0.039	0.046	0.040	0.041	0.047
	4	0.054	0.053	0.049	0.054	0.052
	8	0.041	0.045	0.042	0.047	0.053
	16	0.053	0.058	0.048	0.055	0.053
	32	0.046	0.047	0.033	0.051	0.056
rho=6, Rare+Common variants	0	0.044	0.046	0.049	0.058	0.059
	4	0.054	0.057	0.059	0.056	0.048
	8	0.045	0.050	0.041	0.058	0.057
	16	0.051	0.055	0.050	0.057	0.053
	32	0.047	0.045	0.041	0.051	0.048
